# The effect of moderate gestational alcohol consumption during pregnancy on speech and language outcomes in children: a systematic review

**DOI:** 10.1186/2046-4053-3-1

**Published:** 2014-01-02

**Authors:** Linda M O’Keeffe, Richard A Greene, Patricia M Kearney

**Affiliations:** 1 National Perinatal Epidemiology Centre, Department of Obstetrics and Gynecology, Cork University Maternity Hospital, Cork, Ireland; 2 Department of Epidemiology and Public Health, 4th Floor, Western Gateway Building, University College Cork, Cork, Ireland

**Keywords:** Low, Moderate, Alcohol, Pregnancy, Language, Speech

## Abstract

**Background:**

Consensus has not been reached on safe alcohol consumption recommendations during pregnancy. The National Institutes for Care and Health Excellence (NICE) in the UK suggest that one to two drinks not more than twice per week is safe. However, the speech and language effects of even low levels of alcohol use among offspring are unknown. The aim of this study was to review systematically the evidence on studies of the effect of low to moderate levels of alcohol consumption during pregnancy (up to 70 grams of alcohol per week) compared to abstinence on speech and language outcomes in children.

**Methods:**

Using medical subject headings, PubMed, Web of knowledge, Scopus, Embase, Cinahl and the Cochrane Library were searched from their inception up to March 2012. Case control and cohort studies were included. Two assessors independently reviewed titles, abstracts and full articles, extracted data and assessed quality.

**Results:**

A total of 1,397 titles and abstracts were reviewed of which 51 full texts were retrieved. Three cohort studies totaling 10,642 women met the inclusion criteria. All three studies, (United States (2) and Australia (1)) indicated that language was not impaired as a result of low to moderate alcohol consumption during pregnancy. Two studies were judged to be of low quality based on a six-item bias classification tool. Due to heterogeneity, results could not be meta-analyzed.

**Conclusion:**

Studies included in this review do not provide sufficient evidence to confirm or refute an association between low to moderate alcohol use during pregnancy and speech and language outcomes in children. High quality, population based studies are required to establish the safety of low to moderate levels of alcohol use such as those set out by the NICE guidelines in the UK.

## Background

Speech and language delays in infants and children occur when speech and language abilities are below that expected for a child’s chronological age, while still following the expected developmental sequence [1]. Speech and language development is known to be an important overall developmental milestone in children and early speech and language delays can result in poorer educational outcomes and longer term adverse cognitive and behavioral outcomes throughout the life course [2]. Although the prevalence of speech and language disorders depends on their exact classification and definition, recent studies suggest that some communication disorders may be as high as 13% in primary and secondary school children [3,4].

Research on the predictors of late language emergence at 24 months in an Australian cohort have illustrated the complexity of the predictors of language emergence in the general population showing strong neurobiological and genetic causal mechanisms that operate across a wide variation in maternal and family characteristics [5]. Speech and language impairments are often one of the key features of neurologic damage in children with diagnoses of Fetal Alcohol Spectrum Disorders (FASD) which are as high as 5% in some regions of the United States and Europe [6-8]. However, while the impact of alcohol consumption during pregnancy on birth outcomes [9], mental development [10] and neuropsychological outcomes [11] is well investigated, the effect of alcohol consumption during pregnancy on speech and language development especially at lower or moderate levels is unknown [12].

In Denmark, the Netherlands, Australia, the United Kingdom, Ireland and the United States a number of large population-based longitudinal and cross sectional studies have estimated that between 12% and 81% of babies may be exposed to alcohol during gestation due to maternal alcohol consumption [13-20]. While most women who consume alcohol during pregnancy do so at low or moderate levels, the exact consequences of these levels of alcohol on fetal growth and development have not been established. Recent reviews have suggested that moderate drinking may not be harmful to birth weight, length of gestation or size for gestational age [9,21]. However, evidence suggests that low to moderate alcohol use in pregnancy can still produce functional damage to the brain leading to adverse cognitive and neurological development without obviously affecting other systems such as growth [12]. Specifically, magnetic resonance imaging (MRI) studies have shown that prenatal alcohol exposure can impact on many areas of the brain involved in speech and language development, including the corpus collosum [22].

At present in Canada, the United States, Ireland and New Zealand recommendations advise complete abstinence from alcohol during pregnancy due to uncertain evidence on its effect on growth and development at lower to moderate levels [23-26]. However, the National Institute for Health and Care Excellence in the United Kingdom suggest that one to two units not more than once or twice per week is safe [27]. Establishing the impact of gestational alcohol exposure on speech and language outcomes in children is an important contribution toward understanding, both in the etiology of adverse speech and language development and in developing consistent and comprehensive clinical and government guidelines internationally around alcohol use during pregnancy.

The aim of this review was to systematically search and appraise available case control and cohort studies on the effect of low to moderate alcohol use during pregnancy compared to abstinence from alcohol during pregnancy on speech and language outcomes in children to age 18 years.

## Methods

### Study eligibility criteria

In line with our study protocol, studies were included if they were case control or cohort studies published any time before 1 March 2012, in the English language in a peer reviewed journal. Studies which reported data on low or moderate alcohol exposure (defined as an average of less than 10 grams per day or 70 grams per week during pregnancy) compared to not drinking during pregnancy were included. Studies which reported on any measure or component of language, speech and communication delay, development or disorder in children up to 18 years were included, for example, acquired language disorders and semantic pragmatic disorders (see Additional file 1). We excluded duplicate publications and studies published only as abstracts. Other cognitive and developmental outcomes and nonverbal language outcomes were excluded. Studies of populations with special developmental needs, such as autism spectrum disorder, were also excluded. This review was not registered with PROSPERO [28].

### Search strategy

**Figure 1:**
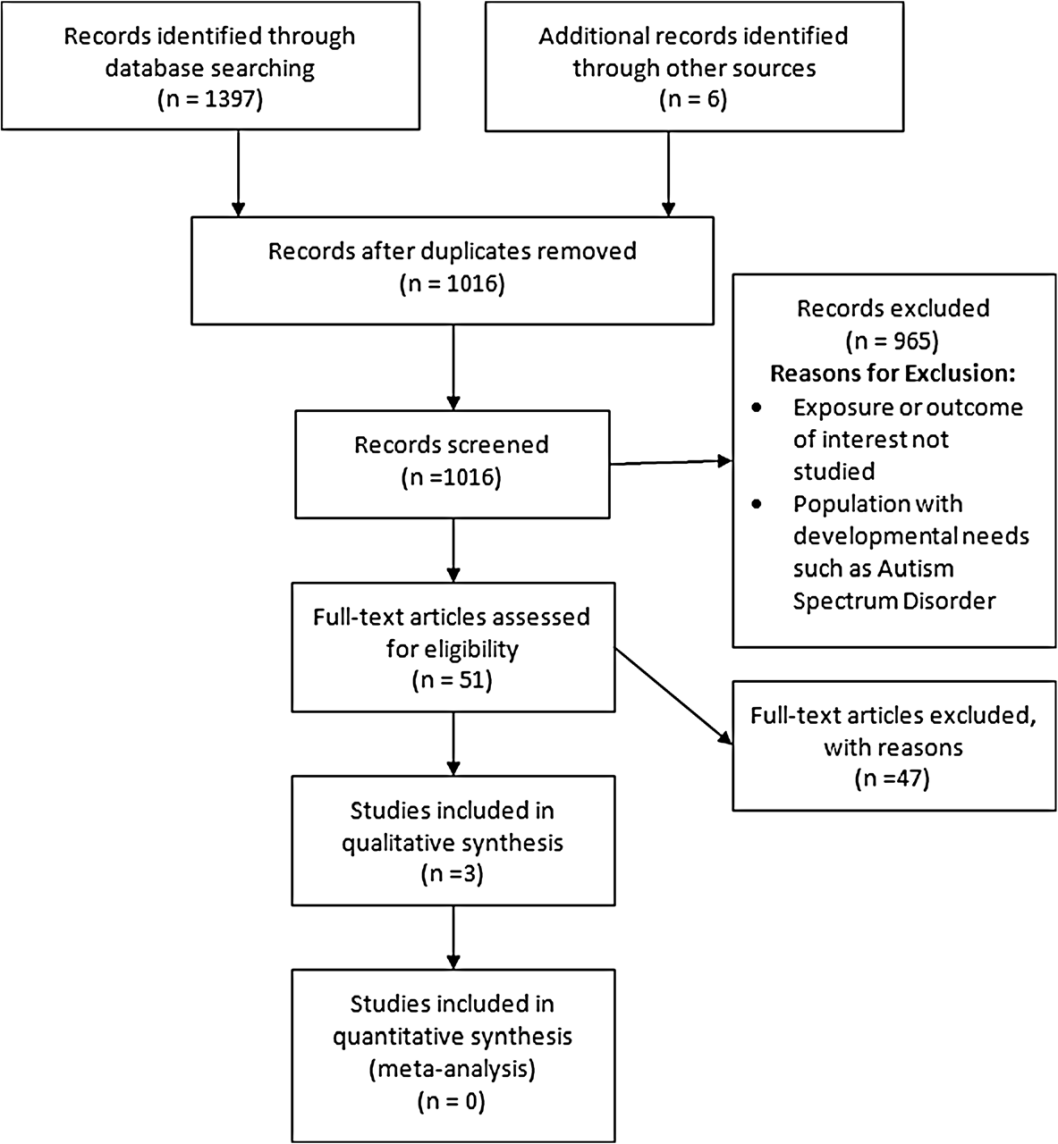
Flow diagram of search strategy.

Embase, PubMed, Cinahl, SCOPUS, Web of Knowledge and The Cochrane Library were searched from their inception up until 1 March 2012 using all appropriate MESH headings related to “alcohol”, “speech and language outcomes”, “pregnancy” and “risk or odds” (see Additional file 1 for detailed search strategy: note that search strategy was not peer reviewed). Search limits of “human” and “female” were applied in PubMed only. All associated MESH terms were combined using “OR”. Finally, all terms were combined using “AND” to yield a total number of abstracts for each database. Reference lists of retrieved articles were also hand searched for further potentially relevant articles. Additionally, the authors of the cohort study by Faden and Graubard [29] were contacted for additional information on their study which was not available in the published paper. All citations were imported to Endnote citation manager and duplicates removed. Figure 1 shows a flow diagram of the results of the search strategy implemented.

### Study and data collection processes

LMOK designed and executed the search strategy (see Additional file 1) which was reviewed by PMK. Two assessors (LMOK, PMK) independently reviewed titles and abstracts of all identified citations. Both reviewers independently evaluated each full text article. Disagreements were resolved by consensus. Articles which were of uncertain relevance were obtained and the full text read. A data extraction form was designed and for the final three articles included in the review, two reviewers independently extracted data on country of origin, years of study, study design, characteristics of participants, exposure definition and ascertainment, outcomes, control for confounders and information on bias (Table 1) as well as available measures of association including odds and risk ratios (Table 2). Due to the heterogeneous nature of the exposure and outcomes across the three studies included in the review, it was not deemed appropriate to conduct a meta-analysis.

**Table 1:** Description of included studies

**Study: study design**	**N**	**Setting**	**Outcome measure: tool used**	**Age outcome recorded**	**Definition of alcohol exposure**	**Exposure ascertainment**	**Confounders included**	**Analyses and measures of effect**
O’Leary *et al.* 2009 [30]: Cohort study	1,739	Western Australia	**Communication delay:** Communication scale from Ages and Stages Questionnaire	Two years	Low = 20 g or less per occasion less than weekly or less than daily	Measured three months after birth for four periods: three months pre-pregnancy and each trimester separately by mail survey	Maternal age, parity, marital status, smoking for each trimester, illicit drug use, depression, anxiety and stress, family factors (antenatal income, presence of partner in household, parenting ability, family functioning)	Odds of language delay by severity of alcohol consumption for each trimester individually
Faden and Graubard 2000 [29]: Cohort study	13,417	USA	**Communication development:** Seven-item language measure of the Denver Developmental Scale	Three years	Mean alcoholic drinks per day during pregnancy	After delivery via a mail survey	None	Mean number of drinks per day for each level of language scale (1 to 7)
Greene *et al*. 1990 [31]: Cohort study	618	Cleveland Metropolitan Hospital, USA	**Communication development:** SICD (Sequenced Inventory of Communication Development)	One, two and three years	Absolute alcohol per day = average number of ounces of alcohol per day reported to have been consumed over all sampled days throughout pregnancy	During pregnancy upon attendance at a clinic	Sex, race, parental education, maternal age, reported usage if street drugs, the day of first antenatal visit, the HOME score, the precise age at testing, the ratings of psychosocial stress and medical problems, maternal IQ	Mean SICD measures adjusted for covariates at selected alcohol levels per day

**Table 2:** Results of included studies

**Author, year**	**Sampling frame**	**Characteristics of mothers**	**N exposed**	**N exposed with outcome**	**N controls**	**N controls with outcome**	**Odds ratios/Mean (95% CI)**
O’Leary *et al*. 2009 [30]	Population-based sample from all women giving birth in Australia 1995 to 1997	**Age**					**Unadjusted**
		<25: 32.7%	Trimester 1: 544	Trimester 1: 79	Trimester 1: 934	Trimester 1: 126	Trimester 1: 0.95 (0.68 to 1.34)
		25 to 29: 13.0%	Trimester 2: 609	Trimester 2: 73	Trimester 2: 946	Trimester 2: 123	Trimester 2: 0.88 (0.63 to 1.23)
		>30: 54.5%	Trimester 3: 665	Trimester 3: 77	Trimester 3: 870	Trimester 3: 113	Trimester 3: 0.83 (0.6 to 1.17)
		**Education**					**Adjusted**
		<12 years: 40.6%					Trimester 1: 0.97 (0.65 to 1.43)
		>12 years: 24.4%					Trimester 2: 0.87 (0.59 to 1.28)
		Degree\Diploma\Trade: 34.8%					Trimester 3: 0.84 (0.57 to 1.23)
		**Smoking**					**Explanation:** Data show unadjusted and confounder adjusted odds ratios for the probability of language delay among low drinkers compared to women who are abstinent at the same time point. All results show reduced odds among low drinkers but results are not statistically significant as indicated by the confidence intervals which span the null value of an odds ratio equal to 1.
		Pre-pregnancy: 27.0%					
		**Parity**					
		Primiparous: 29.8% 1: 30.2%					
		>2: 40.0%					
		**Marital status**					
		Married: 79.2%					
		Cohabiting: 15.4%					
		Single: 5.4%					
		**Illicit Drug use:** 12.5%					
Faden and Graubard 2000 [29]	Population based sample from national birth certificate in the United States	NR*	NR*	Language Outcome (Low to High)	NR*	NR*	**Mean (95% CI)**
				1/7 = 136			0/7 = 0.47 (0.37)
				2/7 = 267			1/7 = 1.64 (1.28)
				3/7 = 493			2/7 = 0.23 (0.10)
				4/7 = 855			3/7 = 0.57 (0.17)
				5/7 = 1323			4/7 = 0.58 (0.12)
				6/7 = 1969			5/7 = 0.57 (0.09)
				7/7 = 2851			6/7 = 0.74 (0.14)
							7/7 = 0.65 (0.10)
							**Explanation:** Data show mean number of drinks per day for scores of 0 to 7 (low to high levels of language development) on the seven-point Denver Language Development scale.
Greene *et al*. 1990 [31]	Hospital based	**Race Black:** 31.5%	179 at 1 year	**1 year**	1 year: 93	1 year: 93	**Expressive Language**
		**Age (mean (SD)):** 22.1 (4.4)		1/3 drink per day:139			**Mean (95% CI)**
		**Parent education in years (mean (SD)):** 10.8 (1.4)		>1/3 to 1.5 drinks per day: 40			1 year
		**Cigarette use in pregnancy (mean (SD)):** 13.8 (12.2)	142 at 2 years	**2 years**	2 year: 94	2 year: 94	A) 25.5 (25.0 to 26.5)
		**Prenatal marijuana:**35.1%		**l2 years**			B) 26.0 (25.0 to 27)
		**Prenatal street drugs: **9.2%		1/3 drink per day:136			2 year
				1/3 to 1.5 drinks per day: 36			A) 30.0 (28.5 to 31.0)
			171 at 3 years	**3 years**	3 year: 92	3 year: 92	B) 29.0 (27.0 to 32.0)
				1/3 drink per day: 132			3 years
				1/3 to 1.5 drinks per day: 39			A) 30.0 (28.0 to 32.0)
							**Receptive language**
							B) 31.0 (28.0 to 34.0)
							1 year
							B) 24.0 (22 to 25)
							A) 24.0 (23 to 25)
							2 years
							A) 39.0 (37.0 to 40.0)
							B) 38.0 (36.0 to 40.0)
							3 years
							A) 24.0 (23.0 to 25.0)
							B) 25.0 (23.0 to 27.0)
							**Explanation:** Mean age-adjusted SICD scores for Expressive and Receptive language development at 1, 2 and 3 years for A) 1/3 drink per day and B) >1/3 to 1 and 1/2 drinks per day compared to abstinence.

*Not reported.

### Quality assessment

The quality of included studies was assessed by evaluating six types of bias to include a rapid assessment of selection, exposure, outcome, confounding, analytical and attrition biases (Table 3) developed by McDonald and colleagues [32] (see Additional file 2). In line with these pre-specified definitions of bias, selection bias was deemed ‘minimal’ if studies reported sampling from a general population of pregnant women rather than a select group. Exposure and outcome assessment were ‘minimal’ if obtained from direct questioning of the mother on the exposure or by using a clearly validated instrument for measuring speech and language outcomes. Confounding bias was assessed as ‘high’ if no confounders were matched or controlled for and ‘minimal’ if at least basic demographic and other key confounders, such as the home environment and parenting variables, were addressed. Analytic bias was deemed ‘moderate’ if no sample size calculation was reported and only a subsample studied, and ‘high’ if inappropriate analyses were undertaken such as multiple unspecified *a priori* or inappropriate subgroup analyses. Attrition was ‘high’ if >20% were lost to follow-up without explanation of the causes.

**Table 3:** Quality assessment based on evaluation of bias

**Author, year**	**Selection bias**	**Exposure bias**	**Outcome assessment bias**	**Confounding factor bias**	**Analytical bias**	**Attrition bias**	**Overall likelihood of bias based mainly on selection and confounding**
O’Leary *et al.*2009 [30]	Minimal (sample selected from a general population rather than a select group)	Low (indirect assessment (postal survey, mailed question))	Minimal (direct question to mother)	Minimal (assessed for common confounders)	Minimal (analyses appropriate for type of sample (if matched))	Moderate (11 to 20% attrition but reasons for loss to follow-up not explained)	Minimal
Faden and Graubard 2000 [29]	Minimal (sample selected from general population, Eligibility Criteria explained)	Low (indirect assessment (postal survey, mailed question))	Minimal (direct question to mother)	High (not assessed for confounders)	Low (analyses not accounting for common statistical adjustment and sample size calculation not performed but all eligible patients studied)	Moderate (11 to 20% but reasons for loss to follow-up not explained)	High
Greene *et al*. 1990 [31]	Moderate (sample selection ambiguous but may be representative)	Minimal (direct questioning (interview) or completion of survey by mother at the time of exposure or close to time of exposure)	Minimal (direct question to mother)	Minimal (assessed for common confounders)	Low (analyses not accounting for common statistical adjustment, power calculation performed)	High (>20% attrition but reasons for loss to follow-up not explained)	Moderate

## Results

### Search results

Overall, 1,397 abstracts were identified of which 387 duplicates were removed (Figure 1). Of the 1,010 articles remaining, 45 full text articles were retrieved. A further six citations were identified through hand searching of full texts leading to a total of 51 studies to undergo full review. Both assessors (LMOK, PMK) independently reviewed all 51 full texts in accordance with inclusion and exclusion criteria and disagreements were settled by consensus. The main reasons for exclusion were lack of reporting on the exposure and outcome of interest and exclusion due to study of alcohol in populations with other developmental disorders such as autism spectrum disorder. Three cohort studies were included, totaling 10,642 women. A summary of the study characteristics and results is shown in Tables 1 and 2.

### Description of included studies

Two studies were from the United States and one study was from Australia. One study collected data on alcohol exposure during pregnancy through direct face to face interviews [31] while the remaining two studies collected exposure status after pregnancy through a postal survey [29,30]. O’Leary *et al*. [30] collected data on dose, pattern and timing of exposure for the three months before pregnancy and each trimester separately approximately 12 weeks after delivery. Faden and Graubard [29] collected data on mean alcohol drinks per day during pregnancy through a postal survey after pregnancy, while Greene *et al*. [31] collected data on the average number of ounces of alcohol per day reported to have been consumed over all sampled days throughout pregnancy by a face to face interview during pregnancy. All three studies used different validated instruments to measure language delay including the parent reported Ages and Stages Questionnaire [30], a parent reported seven-item Language Scale from the Denver Developmental Scale [29] and the Sequenced Inventory of Communication Development (SICD) at one, two and three years [31], a standardized procedure directly administered in the home of the child to diagnose developmental delay in the acquisition and use of language. Only two of the three studies (O’Leary *et al*. [30] and Greene *et al*. [31]) controlled for relevant confounders in their analyses and effect measures were also significantly heterogeneous. O’Leary *et al*. [30] reported crude and adjusted odds ratios while estimates by Faden and Graubard [29] reported mean number of drinks per day for each level of the seven-item Language Scale used. Greene *et al*. [31] reported mean SICD measures for selected levels of absolute alcohol per day at one, two and three years for expressive and receptive language outcomes.

### Systematic review results

Table 3 describes the results of included studies. O’Leary *et al*. [30], retrospective cohort in 1,759 largely married, educated and non-indigenous Australian women, defined low alcohol consumption during pregnancy as 20 g or less per occasion or less than weekly. For low drinking in the first trimester, the odds of language delay were 0.97 (95% confidence interval (CI) 0.65 to 1.43) indicating that low drinkers were 3% less likely to have children with language delay compared to abstinent mothers though the results were not statistically significant as indicated by the wide confidence intervals that span the null value (odds ratio of 1). For low drinking in the second trimester the odds of language delay were 0.87 (95% CI 0.59 to 1.28) and for the third trimester, 0.84 (95% CI 0.57 to 1.23) which also indicated slightly reduced odds of language delay in low drinkers (13% and 16%, respectively) that were not statistically significant as indicated by the confidence intervals spanning the null value. Estimates were adjusted for a range of confounders, including maternal age, parity, marital status, smoking for each trimester, illicit drug use, depression, anxiety and stress, family factors (antenatal income, presence of partner in household, parenting ability, family functioning).

Faden and Graubard [29] retrospective cohort study of 8,885 women reported to have been sampled representatively from the national birth certificate in the United States but did not report socio-demographic and health characteristics of participants. The response rate upon first recruitment to the study was 74% with 83% of the original cohort completing the three-year follow-up. Stratified sampling by both ethnicity and birth weight was conducted. A seven-item language scale was used to measure language development. Potential scores on the scale ranged from 1 to 7 with a lower score indicating lower levels of language development. Mean alcoholic drinks per day during pregnancy were reported for each level of the seven-item language scale. Covariates were not adjusted for and the results reported did not show any dose–response relationship between lower levels of language development and mean number of drinks per day.

The prospective cohort study of 359 mother-infant pairs by Greene *et al*. 1990 [31] in women attending a hospital for antenatal care comprised over 50% black with a mean age of 22 and with over 30% reporting use of marijuana during pregnancy. Women who delivered small for gestational age infants or whose infants were admitted to neonatal intensive care were excluded from the study. This group was also socio-economically disadvantaged. The results were reported in mean SICD measures for selected levels of absolute alcohol per day and were adjusted for sex, race, parental education, maternal age, reported usage of street drugs, the day of first antenatal visit, the Home Observation for the Measurement of the Environment (HOME) score, the precise age at testing, the ratings of psychosocial stress, medical problems and maternal IQ. Significant differences in expressive and receptive language development at one, two and three years were not evident for all three measures of alcohol use obtained, including abstinence, one-third of a UK standard drink per day, greater than one-third to one and a half UK standard drinks per day or approximately two UK standard drinks per day.

### Quality assessment

The results of the quality assessment undertaken are shown in Table 3. Attrition bias was present in all three studies and may have significantly impacted results. In addition, selection, confounding and analytic bias were also present. In the study by Greene *et al*. 1990 [31], selection bias was deemed to be ‘moderate’ due to ambiguous sample selection but ‘minimal’ in both other studies. Confounding bias was deemed to be ‘high’ in Faden and Graubard [29] due to lack of control for confounders but ‘minimal’ in both other studies while analytic bias, though present in both Faden and Graubard [29] and Greene *et al*. [31], was relatively low.

## Discussion

In this systematic review we sought to determine the effect of low to moderate alcohol exposure during pregnancy on speech and language outcomes in children. Poorer language development was not observed in infants exposed to low or moderate alcohol levels [30,31] or was not associated with mean number of drinks per day [29]. However, studies were methodologically heterogeneous and two studies (Faden and Graubard [29] and Greene *et al*. [31]) had a number of limitations including confounding, attrition and selection biases which reduce their validity. Although our search is now 18 months old, due to the low number of articles yielded in our original search of six large international health databases since their inception, we believe our review retains its validity, importance and timeliness.

The method of exposure ascertainment has an impact on the accuracy and validity of reporting of exposure and subsequently the direction of the associations detected. In the studies included in this review, two studies used retrospective ascertainment of exposure while one used concurrent collection. Retrospective data collection used by Faden and Graubard [29] and O’Leary *et al*. [30] is suggested to be subject to higher risk of recall bias and differential misclassification of exposure status whereas concurrent data used by Greene *et al*. [31] are shown to yield more valid information when examining neurodevelopmental outcomes [33]. Alternately, self-administered questionnaires used by both O’Leary *et al*. [30] and Faden and Graubard [29] have been shown to obtain more truthful responses in relation to socially undesirable behaviors, such as alcohol use during pregnancy, than face to face interviews which were employed by Greene and colleagues [34]. In relation to outcome measurement, three different standardized, validated measurement tools were used which cover different domains or aspects of language development and at different ages in three ethnically and culturally diverse populations. Consequently, the presence of adverse effects on speech and language outcomes in children exposed to low to moderate alcohol levels in pregnancy remains difficult to determine from the evidence included in this review due to their heterogeneous populations and exposure and outcome ascertainment.

The presence of attrition and selection bias is likely to have biased reported results toward the null in all studies and explain the lack of a reported association between moderate alcohol use in pregnancy and language delay. In the Australian cohort [30], only 85% of women who agreed to participate originally did so at year two when language outcome was being measured. However, this study had many other strengths and a low level of bias which may have counteracted this effect. Similarly, of the 74% who originally responded to the survey in the cohort studied by Faden and Graubard [29], only 83% completed the three-year follow-up. As participation may be associated with alcohol status and child neurodevelopmental functioning, an apparent association could be masked if children with speech and language problems were systematically lost to follow-up in these studies [35]. For Greene *et al*. [31] selection bias may have also influenced the reported findings. In particular, children with a gestational age under 37 weeks or with admission to neonatal intensive care were excluded from the study. This may have led to the exclusion of children who had a higher risk of language delay or a different pattern of alcohol exposure to other participants.

### Implications for practice

MRI studies have shown that prenatal alcohol exposure can impact upon a number of regions of the brain involved in verbal communication development, such as the corpus collosum, by displacing it, producing shape variability or reducing volume, area and length [22]. Though a lack of association between moderate alcohol use during pregnancy and speech and language development in infants is not implausible, given the low number of studies conducted, the inherent problems of accurate alcohol measurement during pregnancy and lack of biological plausibility with pathophysiological evidence, further research is required. In particular, future studies which more robustly assess the impact of alcohol use on speech and language outcomes due to the likely impact of attrition, selection and confounding biases are required. Losses to follow-up were considerable threats to validity in studies reviewed. Efforts to account for both participation bias which occurs upon recruitment to studies and attrition bias occurring later in the study should be incorporated in future studies of this nature. The complex nature of the development of speech and language delays including the interaction of environmental, neurodevelopmental and familial factors [5] as well as the underlying cultural and ethnic differences that may result in varying attitudes, practices and norms relating to alcohol and speech and language outcomes must be addressed [16]. Consequently, until, large scale, population-based, longitudinal studies of gestational alcohol use and speech and language outcomes emerge, healthcare providers should advise women to abstain from consuming alcohol during pregnancy while policy makers should remain aware of limited research evidencing safe alcohol consumption thresholds during pregnancy.

## Conclusions

International guidelines have not reached consensus on safe alcohol recommendations for pregnant women. The findings of this review reveal the dearth of research on the effect of low to moderate gestational alcohol use and speech and language outcomes in children. Future research should carefully address the validity and accuracy of exposure and outcome ascertainment and pay particular attention to reducing the risk of selection, attrition and confounding biases. Healthcare providers should continue to advice abstinence from alcohol during pregnancy until further evidence on the effect of low-moderate gestational alcohol use becomes available. Policy makers should remain aware of limited research showing the safety of alcohol use in pregnancy in relation to childhood development such as speech and language outcomes.

## Abbreviations

MRI: Magnetic resonance imaging; NICE: National Institutes for Health and Care Excellence; NR: Not reported; SICD: Sequenced Inventory of Communication Development; HOME: Home Observation for Measurement of the Environment.

## Competing interests

The authors have no conflicts of interest to disclose.

## Authors’ contributions

LMO’K conceptualized and designed the study, devised and conducted the search strategy, drafted the initial manuscript, and approved the final manuscript as submitted. RAG contributed to the concept and design of the study, reviewed and revised the manuscript, and approved the final manuscript as submitted. PMK supervised the methods, critically reviewed the manuscript, and approved the final manuscript as submitted. All authors read and approved the final manuscript.

## Authors’ information

Linda Marie O’Keeffe is a PhD candidate at University College Cork, Ireland. Her PhD thesis focuses on examining the growth and cognitive impact of alcohol use during pregnancy. This topic is important and timely because significant controversy exists around the safety of low levels of alcohol consumption during pregnancy. Secondly, the prevalence of alcohol use during pregnancy remains high in many Western European countries, such as Ireland (in excess of 50%), despite the absence of a threshold below which harm to the fetus does not occur. Conducting systematic reviews of the effect of low levels of alcohol use on cognitive and developmental outcomes in childhood is an important part of assessing comprehensively fetal alcohol effects beyond birth.

## Supplementary Material

Additional file 1 Sample Search Strategy. Click here for file

Additional file 2 Quality Assessment Tool. Click here for file

## References

[B1] Law J Boyle J Harris F Harkness A Screening for speech and language delay: a systematic review of the literature Health Technol Assess 1998 2 1 184 9728296

[B2] Nelson HD Nygren P Walker M Panoscha R Screening for speech and language delay in preschool children: systematic evidence review for the US preventive services task force Pediatrics 2006 117 e298 e319 10.1542/peds.2005-1467 16452337

[B3] McLeod S Harrison LJ Epidemiology of speech and language impairment in a nationally representative sample of 4- to 5-year-old children J Speech Lang Hear Res 2009 52 1213 10.1044/1092-4388(2009/08-0085) 19403947

[B4] McLeod S McKinnon DH Prevalence of communication disorders compared with other learning needs in 14,500 primary and secondary school students Int J Lang Commun Disord 2007 42 Suppl 1 37 59 17454236 10.1080/13682820601173262

[B5] Hawkins SS Stern AD Gillman MW Do state breastfeeding laws in the USA promote breast feeding? J Epidemiol Community Health 2013 67 250 256 10.1136/jech-2012-201619 23087383 PMC3574222

[B6] Mattson SN Crocker N Nguyen TT Fetal alcohol spectrum disorders: neuropsychological and behavioral features Neuropsychol Rev 2011 21 81 101 10.1007/s11065-011-9167-9 21503685 PMC3410672

[B7] May PA Gossage JP Kalberg WO Robinson LK Buckley D Manning M Hoyme HE Prevalence and epidemiologic characteristics of FASD from various research methods with an emphasis on recent in-school studies Dev Disabil Res Rev 2009 15 176 192 10.1002/ddrr.68 19731384

[B8] May PA Fiorentino D Phillip Gossage J Kalberg WO Eugene Hoyme H Robinson LK Coriale G Jones KL del Campo M Tarani L Romeo M Kodituwakku PW Deiana L Buckley D Ceccanti M Epidemiology of FASD in a province in Italy: prevalence and characteristics of children in a random sample of schools Alcohol Clin Exp Res 2006 30 1562 1575 10.1111/j.1530-0277.2006.00188.x 16930219

[B9] Patra J Bakker R Irving H Jaddoe VW Malini S Rehm J Dose–response relationship between alcohol consumption before and during pregnancy and the risks of low birthweight, preterm birth and small for gestational age (SGA)-a systematic review and meta-analyses BJOG 2011 118 1411 1421 10.1111/j.1471-0528.2011.03050.x 21729235 PMC3394156

[B10] Testa M Quigley BM Das Eiden R The effects of prenatal alcohol exposure on infant mental development: a meta-analytical review Alcohol Alcohol 2003 38 295 304 10.1093/alcalc/agg087 12814894

[B11] Flak AL Su S Bertrand J Denny CH Kesmodel US Cogswell ME The association of mild, moderate, and binge prenatal alcohol exposure and child neuropsychological outcomes: a meta‒analysis Alcohol Clin Exp Res 2013 Epub ahead of print 10.1111/acer.12214 23905882

[B12] Gray R Mukherjee RAS Rutter M Alcohol consumption during pregnancy and its effects on neurodevelopment: what is known and what remains uncertain Addiction 2009 104 1270 1273 10.1111/j.1360-0443.2008.02441.x 19215606

[B13] Colvin L Payne J Parsons D Kurinczuk JJ Bower C Alcohol consumption during pregnancy in nonindigenous west Australian women Alcohol Clin Exp Res 2007 31 276 284 10.1111/j.1530-0277.2006.00303.x 17250620

[B14] Kelly Y Sacker A Gray R Kelly J Wolke D Quigley MA Light drinking in pregnancy, a risk for behavioural problems and cognitive deficits at 3 years of age? Int J Epidemiol 2009 38 129 140 10.1093/ije/dyn230 18974425

[B15] Bakker R Pluimgraaff LE Steegers EA Raat H Tiemeier H Hofman A Jaddoe VW Associations of light and moderate maternal alcohol consumption with fetal growth characteristics in different periods of pregnancy: the Generation R Study Int J Epidemiol 2010 39 777 789 10.1093/ije/dyq047 20385669

[B16] Andersen AM Andersen PK Olsen J Grønbæk M Strandberg-Larsen K Moderate alcohol intake during pregnancy and risk of fetal death Int J Epidemiol 2012 41 405 413 10.1093/ije/dyr189 22253313

[B17] Mullally A Cleary BJ Barry J Fahey TP Murphy DJ Prevalence, predictors and perinatal outcomes of peri-conceptional alcohol exposure - retrospective cohort study in an urban obstetric population in Ireland BMC Pregnancy Childbirth 2011 11 27 10.1186/1471-2393-11-27 21481224 PMC3080836

[B18] Royal College of Obstetricians and Gynecologists Alcohol consumption and the outcomes of pregnancy (RCOG Statement No 5) 2006 http://www.rcog.org.uk/womens-health/clinical-guidance/alcohol-consumption-and-outcomes-pregnancy

[B19] McCarthy FP O'Keeffe LM Khashan AS North RA Poston L McCowan LM Baker PN Dekker GA Roberts CT Walker JT Association between maternal alcohol consumption in early pregnancy and pregnancy outcomes Obstet Gynecol 2013 122 830 837 10.1097/AOG.0b013e3182a6b226 24084541

[B20] O'Keeffe LM Kearney PK Greene RA Surveillance during pregnancy: methods and response rates to a hospital based cross sectional study of the pregnancy risk assessment monitoring system in Ireland BMC Pregnancy Childbirth 2013 13 180 10.1186/1471-2393-13-180 24066665 PMC3850906

[B21] Henderson J Gray R Brocklehurst P Systematic review of effects of low–moderate prenatal alcohol exposure on pregnancy outcome BJOG 2007 114 243 252 10.1111/j.1471-0528.2006.01163.x 17233797

[B22] Lebel C Roussotte F Sowell ER Imaging the impact of prenatal alcohol exposure on the structure of the developing human brain Neuropsychol Rev 2011 21 102 118 10.1007/s11065-011-9163-0 21369875 PMC3098972

[B23] Butt P Beirness D Gliksman L Alcohol and Health in Canada: A summary of Evidence and Guidelines for Low Risk Drinking 2011 Ottawa: Canadian Centre on Substance Abuse

[B24] Health Services Executive Alcohol and Pregnancy http://www.yourdrinking.ie/alcohol-and-pregnancy

[B25] Ministry of Health Alcohol and Pregnancy: A Practical Guide for Health Professionals 2010 Wellington: Ministry of Health

[B26] U.S. Department of Health and Human Services U.S. surgeon general releases advisory on alcohol use in pregnancy [http://www.surgeongeneral.gov/news/2005/02/sg02222005.html]

[B27] National Institute for Care and Clinical Excellence (NICE) Antenatal Care: Routine Care for Healthy Pregnant Women: NICE Clinical Guidelines 62 2008

[B28] PROSPERO International prospective register of systematic reviews http://www.crd.york.ac.uk/prospero/

[B29] Faden VB Graubard BI Maternal substance use during pregnancy and developmental outcome at age three J Subst Abuse 2000 12 329 340 10.1016/S0899-3289(01)00052-9 11452837

[B30] O'Leary C Zubrick SR Taylor CL Dixon G Bower C Prenatal alcohol exposure and language delay in 2-year-old children: the importance of dose and timing on risk Pediatrics 2009 123 547 554 10.1542/peds.2008-0459 19171621

[B31] Greene T Ernhart CB Martier S Sokol R Ager J Prenatal alcohol exposure and language development Alcohol Clin Exp Res 1990 14 937 945 10.1111/j.1530-0277.1990.tb01842.x 2088132

[B32] McDonald SD Han Z Mulla S Murphy KE Beyene J Ohlsson A Preterm birth and low birth weight among *in vitro* fertilization singletons: a systematic review and meta-analyses Eur J Obstet Gynecol Reprod Biol 2009 146 138 10.1016/j.ejogrb.2009.05.035 19577836

[B33] Jacobson SW Chiodo LM Sokol RJ Jacobson JL Validity of maternal report of prenatal alcohol, cocaine, and smoking in relation to neurobehavioral outcome Pediatrics 2002 109 815 10.1542/peds.109.5.815 11986441

[B34] Bloomfield K Hope A Kraus L A review of alcohol survey methodology: towards a standardised measurement instrument for Europe Drug: Educ, Prev, Policy 2011 http://www.alcsmart.ipin.edu.pl/files/prop_01.pdf

[B35] Kesmodel U Bertrand J Støvring H Skarpness B Denny C Mortensen E The effect of different alcohol drinking patterns in early to mid pregnancy on the child’s intelligence, attention, and executive function BJOG 2012 119 1180 1190 10.1111/j.1471-0528.2012.03393.x 22712700 PMC4435537

