# Supplementary material for: The effect of moderate gestational alcohol consumption during pregnancy on speech and language outcomes in children: a systematic review
Source: Syst Rev. 2014 Jan 2;3:1. doi: 10.1186/2046-4053-3-1 (PMC3892059; doi:10.1186/2046-4053-3-1)
Supplement: Additional file 1 — Sample Search Strategy. [file 2046-4053-3-1-S1.doc]

| **Appendix 1 Sample Search Strategy [repeated for all databases]** | |
| --- | --- |
| Search terms | **No of records returned** |
| *For PUBMED until 01/03/2012 LIMIT: humans, female* |  |
| 1. Alcohol | 15383 |
| 1. Alcoholic beverages | 3389 |
| 1. Absolute alcohol | 17689 |
| 1. Alcohol, absolute | 17689 |
| 1. Light drinking | 1089 |
| 1. “light drinking” | 87 |
| 1. Moderate drinking | 2917 |
| 1. “moderate drinking” | 284 |
| 1. ethanol | 17198 |
| 1. Alcohol Consumption | 37384 |
| 1. Alcohol drinking | 30355 |
| 1. Consumption, Alcohol | 37384 |
| 1. Drinking, Alcohol | 30355 |
| 1. Behavior, Drinking | 28513 |
| 1. Behaviors, Drinking | 28757 |
| 1. Drinking Behaviors | 28766 |
| 1. Alcohol use | 72993 |
| 1. Alcohol intake | 34594 |
| 1. [#1 or #2 or #3 or #4 or #5 or #6 or #7 or #8 or #9 or #10 or #11 or #12 or #13 or #14 or #15 or #16 or #17 or #18] | 156757 |
| 1. Language | 66816 |
| 1. Languages | 67439 |
| 1. Communication | 124437 |
| 1. Personal, communication | 5181 |
| 1. Communication, personal | 124437 |
| 1. Speech | 34732 |
| 1. Verbal behavior | 19320 |
| 1. Behavior, verbal | 19300 |
| 1. Behaviors, verbal | 19513 |
| 1. Verbal behaviors | 19520 |
| 1. Speech intelligibility | 2159 |
| 1. Intelligibilities, speech | 2164 |
| 1. Intelligibility, speech | 2164 |
| 1. Speech intelligibilities | 2164 |
| 1. Language development disorder | 4944 |
| 1. Development disorder, language | 4944 |
| 1. Speech or language, developmental disorder | 36110 |
| 1. Language disorders, developmental | 5162 |
| 1. Developmental disorder, speech or language | 67159 |
| 1. Developmental language disorders | 5162 |
| 1. Developmental language disorder | 5052 |
| 1. Language disorder, developmental | 5052 |
| 1. Speech delay | 5360 |
| 1. Delay, speech | 5360 |
| 1. Speech delays | 4832 |
| 1. Semantic-pragmatic disorder | 4655 |
| 1. Semantic-pragmatic disorders | 4671 |
| 1. Auditory processing disorder, central | 4743 |
| 1. Central auditory processing disorder | 4743 |
| 1. Language delay | 5319 |
| 1. Language delays | 4890 |
| 1. Communication disorder | 29976 |
| 1. Communication disorders | 28839 |
| 1. Communicative disorders | 29630 |
| 1. Communicative disorder | 29002 |
| 1. Childhood communication disorders | 28839 |
| 1. Childhood communication disorder | 28892 |
| 1. Communication disorder, childhood | 28892 |
| 1. Communication disorders, childhood | 28839 |
| 1. Neurogenic communication disorders | 28839 |
| 1. Communication disorder, neurogenic | 28843 |
| 1. Neurogenic communication disorder | 28843 |
| 1. Communication disorders, neurogenic | 28839 |
| 1. Communication disorders, development | 6355 |
| 1. Communication disorders, developmental | 28839 |
| 1. Communication disorder, developmental | 28957 |
| 1. Developmental communication disorder | 28957 |
| 1. Developmental communication disorders | 28839 |
| 1. Communicative dysfunction | 28877 |
| 1. Communicative dysfunctions | 28843 |
| 1. Dysfunction, communicative | 28877 |
| 1. Dysfunctions, communicative | 28843 |
| 1. Acquired communication disorders | 28839 |
| 1. Acquired communication disorder | 28856 |
| 1. Communication disorder, acquired | 28856 |
| 1. Communication disorders, acquired | 28839 |
| 1. Communication disabilities | 29343 |
| 1. Communication disability | 29729 |
| 1. Disabilities, communication | 29343 |
| 1. Disability, communication | 29729 |
| 1. Language development | 12339 |
| 1. Development, language | 12339 |
| 1. Developments, language | 12377 |
| 1. Language developments | 12377 |
| 1. Child language | 15053 |
| 1. Language, child | 15053 |
| 1. Languages, child | 15196 |
| 1. Language disorder | 23187 |
| 1. Language disorder | 24315 |
| 1. Acquired language disorders | 23187 |
| 1. Acquired language disorder | 23215 |
| 1. Language disorder, acquired | 23215 |
| 1. Language disorders, acquired | 23187 |
| 1. [#20 or #21 or #22 or #23 or #24 or #25 or #26 or #27 or #28 or #29 or #30 or #31 or #32 or #33 or #34 or #35 or #36 or #37 or #38 or #39 or #40 or #41 or #42 or #43 or #44 or #45 or #46 or #47 or #48 or #49 or #50 or #51 or #52 or #53 or #54 or #55 or #56 or #57 or #58 or #59 or #60 or #61 or #62 or #63 or #64 or #65 or #66 or #67 or #68 or #69 or #70 or #71 or #72 or #73 or #74 or #75 or #76 or #77 or #78 or #79 or #80 or #81 or #82 or #83 or #84 or #85 or #86 or #87 or #88 or #89 or #90 or #91 or #92 ] | 165250 |
| 1. [#19 AND #93] | 2976 |
| 1. Pregnancy | 519730 |
| 1. Pregnant | 75536 |
| 1. Pregnancies | 522411 |
| 1. Gestation | 524056 |
| 1. Pregnant women | 58051 |
| 1. Women, pregnant | 58051 |
| 1. Pregnant woman | 62435 |
| 1. Woman, pregnant | 63130 |
| 1. Prenatal | 86622 |
| 1. Antenatal | 16192 |
| 1. Gestational | 69955 |
| 1. [#94 or #95 or #96 or #97or #98 or #99 or #100 or #101 or #102 or #103 or #104 or #105] | 545941 |
| 1. [#19 AND #93 AND #106] | 407 |
| 1. Case-control | 121458 |
| 1. Cohort | 174503 |
| 1. Systematic review | 282313 |
| 1. Review | 411414 |
| 1. Prospective | 615054 |
| 1. retrospective | 344299 |
| 1. odds | 107649 |
| 1. risk | 715402 |
| 1. [#108 or #109 or #110 or #111 #112 or #113] | 1724908 |
| 1. [#19 AND #93 AND #106 AND #116] | 243 |
